# The Stabilization Technique for Ankle Lateral Repair: An All‐Inside 2‐Portal Arthroscopic Repair With Knotless Suture Anchors and Suture Tape Augmentation

**DOI:** 10.1002/atn2.70050

**Published:** 2026-08-02

**Authors:** Bruno S. Pereira, Filipe Sá Malheiro, Pieter D’Hooghe

**Affiliations:** ^1^ Clínica Espregueira ‐ FIFA Medical Centre of Excellence Porto Portugal; ^2^ Unidade Local de Saúde de Barcelos/Esposende Barcelos Portugal; ^3^ ICVS/3B's‐PT Government Associate Laboratory Braga, Guimarães Portugal; ^4^ Foot and Ankle Unit, Human Anatomy Unit, School of Medicine University of Barcelona Barcelona Spain; ^5^ Dom Henrique Research Centre Porto Portugal; ^6^ Fortius Clinic London UK

## Abstract

Lateral ankle sprains are among the most frequent injuries encountered in sports, with the anterior talofibular ligament being the structure most often affected. Although many individuals achieve full recovery, a substantial number experience chronic lateral ankle instability, which presents with recurrent sprains, mechanical instability, and ongoing discomfort. Although the open Broström‐Gould repair remains the gold standard, arthroscopic techniques have gained attraction due to their minimally invasive nature, improved visualization, and faster recovery. In this article, we describe an all‐inside arthroscopic technique for anterior talofibular ligament repair using knotless suture anchors with suture tape augmentation. This approach enables precise anatomic repair, reduces complications associated with knot irritation, and provides enhanced biomechanical stability for athletes.

## Graphical abstract

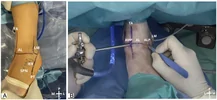

**VIDEO 1:** This video shows a surgical technique for chronic lateral ankle instability. The patient is placed supine with the tourniquet at the proximal thigh. Anatomical reference lines and points are drawn on the skin to help create the classic anterior arthroscopy portals. After the 2 portal incisions, a medial to lateral inspection is conducted with the arthroscope, to evaluate the joint in terms of cartilage, conflicts, and ligamentous structures. Then, it is important to assess the tissue to be repaired. Next, it is prepared to receive the anchoring. Through this, we are preparing the footprints of the ligamentous structures on the fibula—ATFL and CFL. The FiberTak guide is placed, and a bone socket is created, at 1 cm from the distal tip of the fibula, by sliding the guide wire to create a bone tunnel. Keeping the guide in place, the anchor is inserted and impacted with gentle mallet taps until the handle is flush with the guide. The working blue suture with the needle is removed and the needle is cut. Next, the guide and anchor introducer are removed. After that, the wires are slowly pulled on to open the intraosseous anchor and secure it. Posteriorly, the working blue suture tip is placed on the mini scorpion, and, with the mini scorpion inverted, it is passed through the ligament. The next step is based on the work principles of the knotless system. Outside the cannula, the blue wire is passed through the looped end of the black and white loop, which is white with black stripes, to the purple mark on the blue wire. Following that, with a series of light tugs on the loop, the working blue suture is pulled inside the anchor, while simultaneously pulling the ligament into the footprint, with the foot in a neutral position. Lastly, the wire is cut with arthroscopic scissors. The second anchor, a Knotless 2.6 FiberTak with SpeedBridge, is placed below the footprint of the AITFL. It has a component identical to the previous anchor and is able to retrieve the ligament tissue from the superior portion of the ATFL. The SpeedBridge tape passes over the capsule, and a 3.5 SwiveLock anchor is placed anterior to the ATFL footprint on the talus. This fixation should be performed with the ankle in plantar flexion. Video content can be viewed at https://doi.org/10.1002/atn2.70050.

Ankle sprains represent the most common ligamentous injuries in sports and in the general population.^1^ Among these, lateral ankle sprains predominate, with the anterior talofibular ligament (ATFL) as the primary ligament involved in approximately 85% of cases.^2^ Although the majority of patients achieve full recovery, up to 70% may develop chronic lateral ankle instability (CLAI), leading to chronic pain, instability, and mechanical dysfunction.^3^, ^4^

The Broström‐Gould technique has long been considered the standard surgical treatment for CLAI.^5^ However, arthroscopic techniques have emerged as a viable alternative, offering comparable or even superior outcomes with the advantages of reduced surgical morbidity and faster rehabilitation.^6^, ^7^, ^8^, ^9^ Recent innovations include the use of knotless suture anchors, which address complications associated with traditional knotted constructs, such as irritation and soft‐tissue impingement, reported in up to 11.6% of cases.^6^, ^7^, ^8^

This article details a refined arthroscopic technique for anatomic ATFL repair and augmentation using knotless suture anchors and an internal brace. This method allows for a minimally invasive, biomechanically stable reconstruction, particularly suited for athletes, because of the potential for earlier rehabilitation and possibly stronger initial stability.^9^

## SURGICAL TECHNIQUE

### Preoperative Planning and Patient Setup

Surgical indication is based on clinical evaluation, patient history and imaging (radiographs and magnetic resonance imaging) consistent with CLAI (Video 1).

Anesthesia is selected collaboratively, considering patient comorbidities.

**FIGURE 1:**
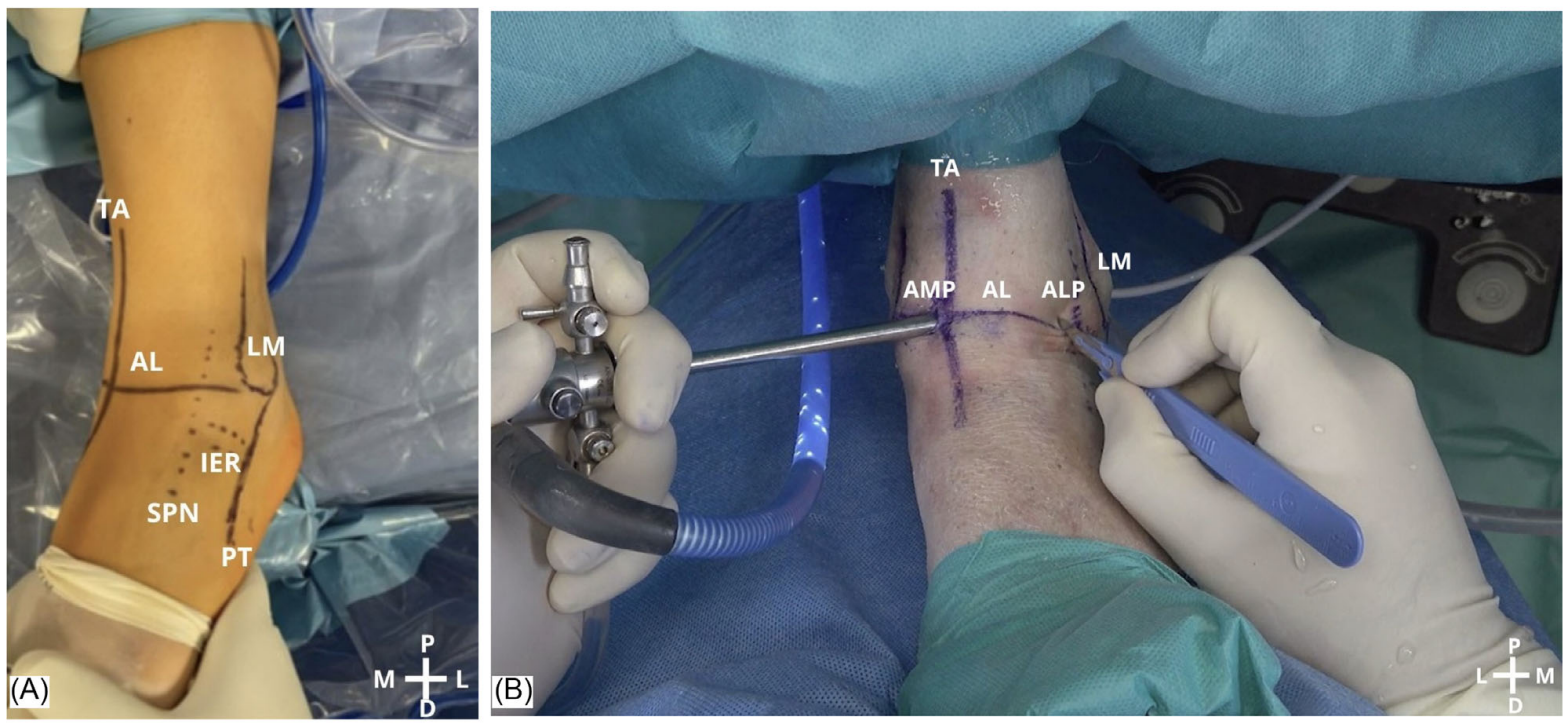
A left ankle is shown with the patient supine. (A) Reference lines and points are drawn to help to create the arthroscopic portals. (B) Conventional anteromedial arthroscopic portal is established and the incision's anterolateral portal is made (Viewing portal: Anteromedial portal). (AL, articular line; ALP, anterolateral portal; AMP, anteromedial portal; D, distal; IER, inferior extensor retinaculum; L, lateral; LM, lateral malleolus; M, medial; P, proximal; PT, peroneal tendon; SPN, superficial peroneal nerve; TA, tibialis anterior tendon.)

The patient is placed supine with a thigh tourniquet. Instability is confirmed via the validated anterior drawer test. Key anatomic landmarks are marked, including the anterior tibial tendon, medial and lateral malleoli, superficial peroneal nerve, and the tibiotalar joint line (Figure 1).

### Portal Placement

Standard anteromedial and anterolateral arthroscopy portals are created (Figure 1). The anteromedial portal is established medial to the tibialis anterior tendon; the anterolateral portal is placed lateral to the peroneus tertius tendon with direct visualization (Table 1). Blunt dissection is used to avoid injury to the neurovascular structures.

**TABLE 1:** Pearls and Pitfalls

Pearls	Pitfalls
Correct identification of the anterior tibial tendon, medial malleolus, lateral malleolus, superficial peroneal nerve, and articular line are important in order to perform the correct skin incision	Not recognizing other anatomical predisposal factors for CLAI may lead to potential failure
Carefully remove damaged tissue and prepare the attachment site to receive the anchoring	When preparing the footprint with shaver, see if it is easy to remove bone
Correct identification of the footprint of ATFL and CFL	Create a bone tunnel at 1 cm from the distal tip of the fibula
Good tissue to be reattached	Grabbing the remaining ligament with a grasper
If it is a soft bone, use a 1.6‐mm drill, and if it is a hard bone use a 1.8‐mm drill	Be careful not to damage the superficial peroneal nerve
Pull gently the anchor wires with small pulls to expend it, and not with one strong single pull, to decrease the probability of an anchor pullout	

ATFL, anterior talofibular ligament; CFL, calcaneofibular ligament; CLAI, chronic lateral ankle instability.

### Diagnostic Arthroscopy and Site Preparation

**FIGURE 2:**
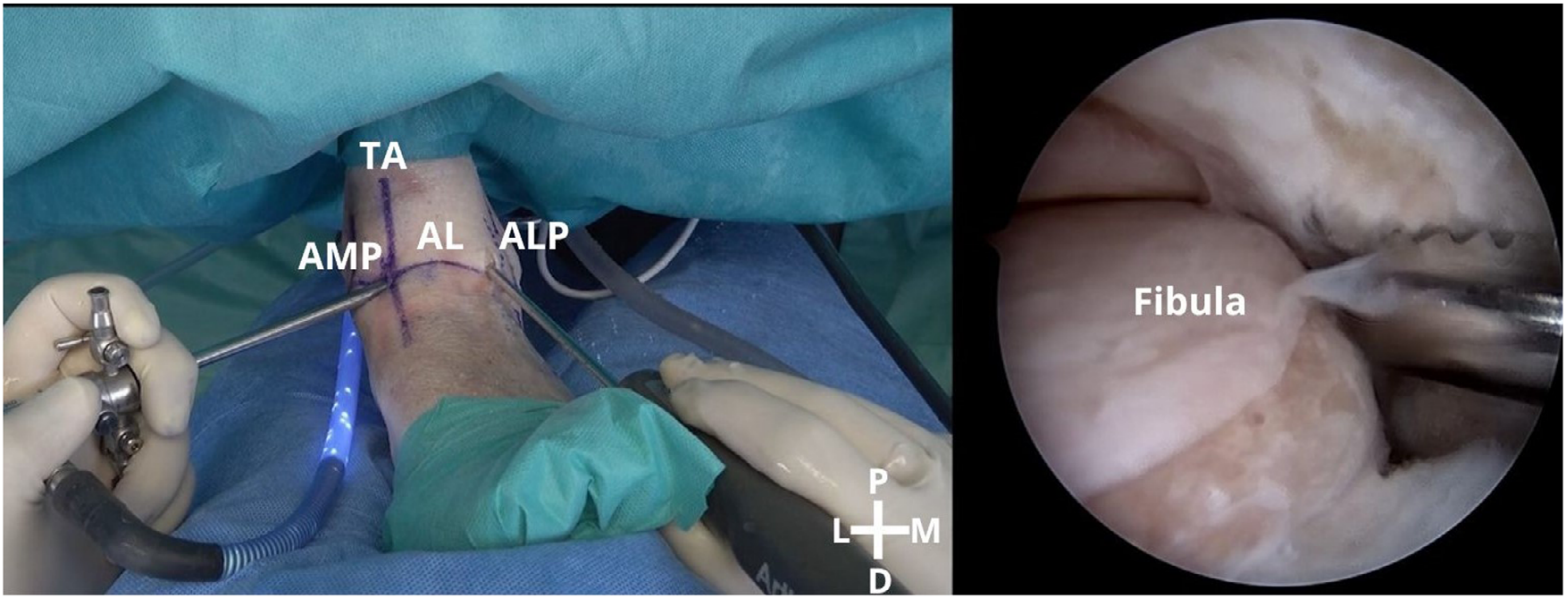
A left ankle is shown with the patient supine. The footprints of the ligamentous structures on the fibula are prepared (Viewing portal: Anteromedial portal). (AL, articular line; ALP, anterolateral portal; AMP, anteromedial portal; D, distal; L, lateral; M, medial; P, proximal; TA, tibialis anterior tendon.)

Initial arthroscopic assessment includes inspection of the cartilage, soft tissue, and ligamentous structures. The ATFL and calcaneofibular ligament footprints on the fibula are identified using a shaver to expose healthy bone and prepare for anchor insertion (Figure 2).

### Anchor Placement and Ligament Reattachment

**FIGURE 3:**
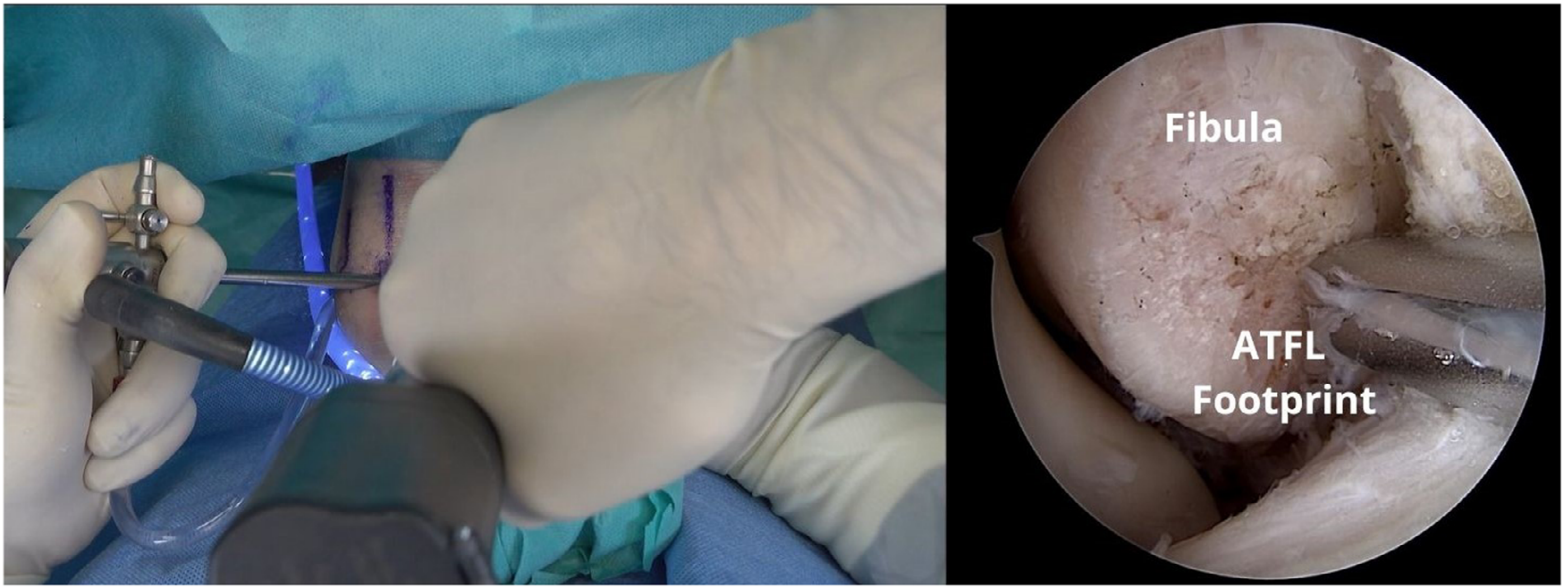
A left ankle is shown with the patient supine. The FiberTak guide is placed, and a bone socket is created, at 1 cm from the distal tip of the fibula, where is the ATFL footprint (Viewing portal: Anteromedial portal). (ATFL, anterior talofibular ligament.)

Using a 1.9‐mm FiberTak guide (Arthrex, Naples, FL), the first bone tunnel is drilled with the guidewire, 1 cm proximal to the fibular tip at the ATFL footprint (Figure 3). For soft bone, a 1.6‐mm guidewire is used, whereas for hard bone, a 1.8‐mm guidewire is used. The drill is passed 2 times in the footprint to prepare the anchorage.

**FIGURE 4:**
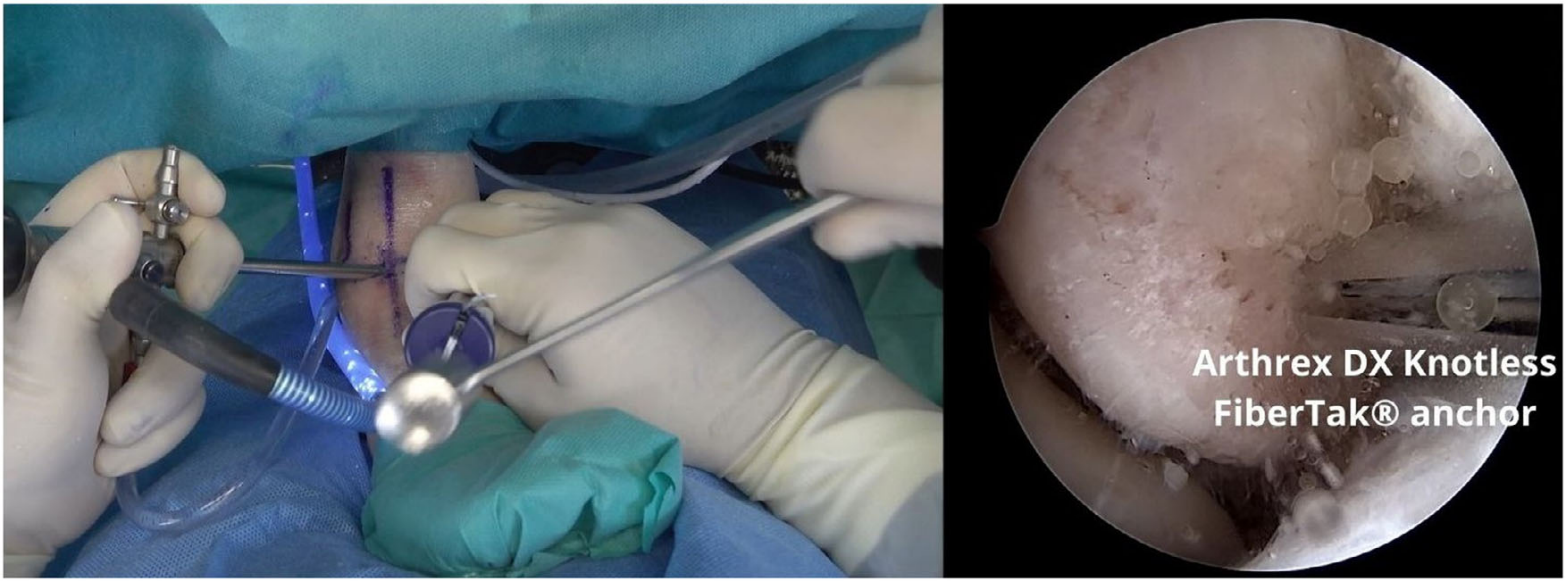
A left ankle is shown with the patient supine. Keeping the guide in place, the anchor (Arthrex DX Knotless FiberTak anchor) is inserted and impacted with tape and hammer (Viewing portal: Anteromedial portal).

**FIGURE 5:**
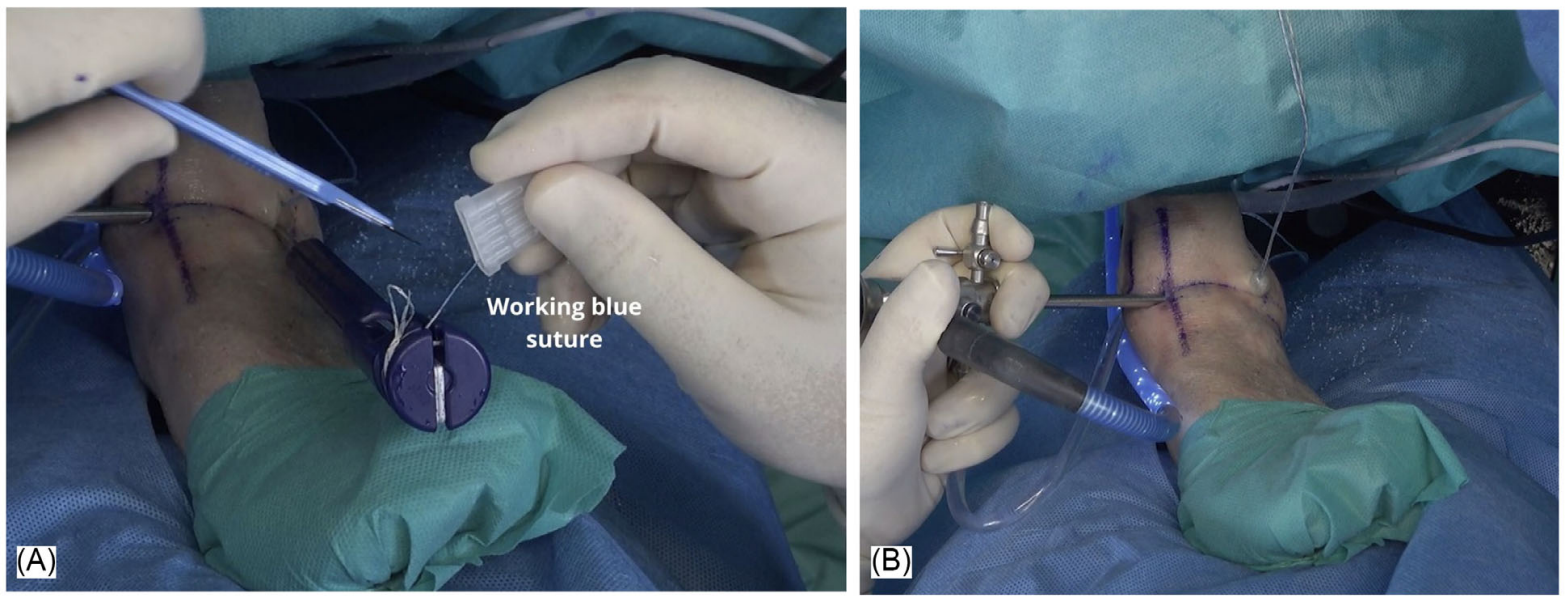
A left ankle is shown with the patient supine. (A) The working blue suture with the needle is removed and the needle is cut. (B) The guide and the anchor introducer are removed (Viewing portal: Anteromedial portal).

**FIGURE 6:**
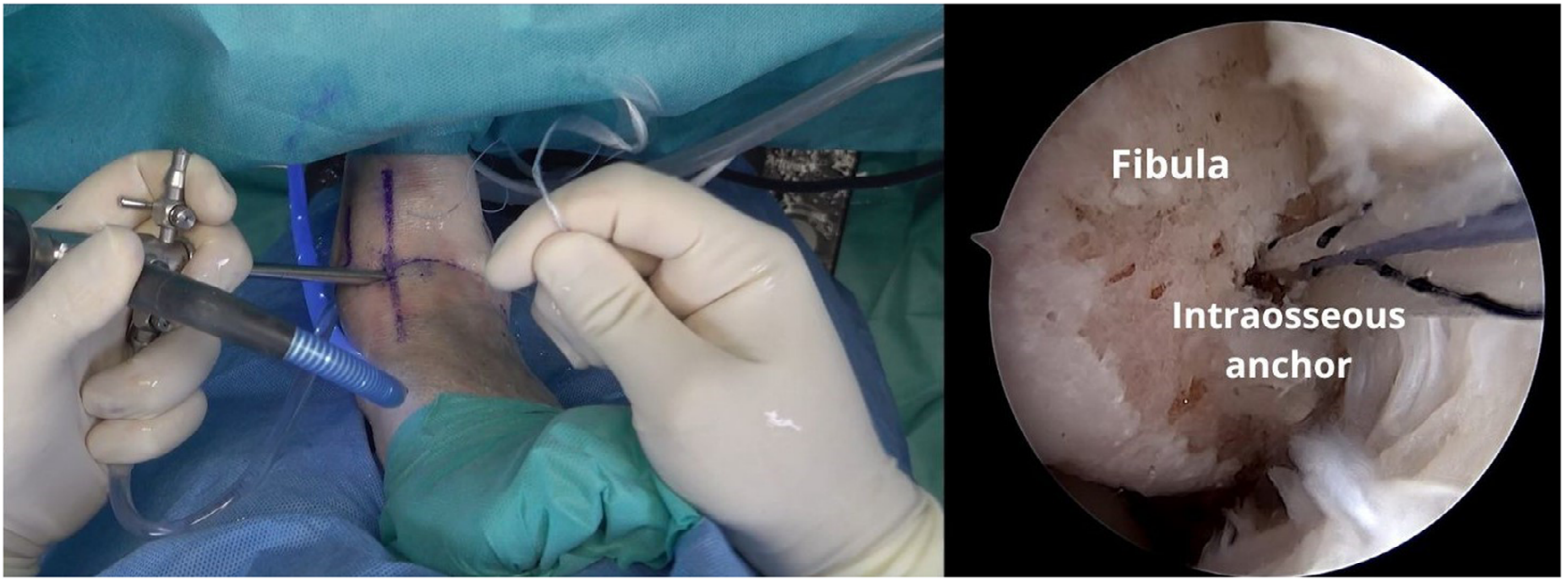
A left ankle is shown with the patient supine. The wires are slowly pulled on to open the intraosseous anchor and secure it (Viewing portal: Anteromedial portal).

The anchor (Arthrex DX Knotless FiberTak anchor) is inserted through the guide and impacted with gentle taps until the handle is flush with the guide (Figure 4). The black rubber ring that secures the wires to release the sutures from the handle is removed. The working blue suture with the needle is removed (Figure 5). The guide and the anchor introducer are removed also (Figure 5). Thereafter, the wires are slowly pulled on to open the intraosseous anchor and secure it (Figure 6).

**FIGURE 7:**
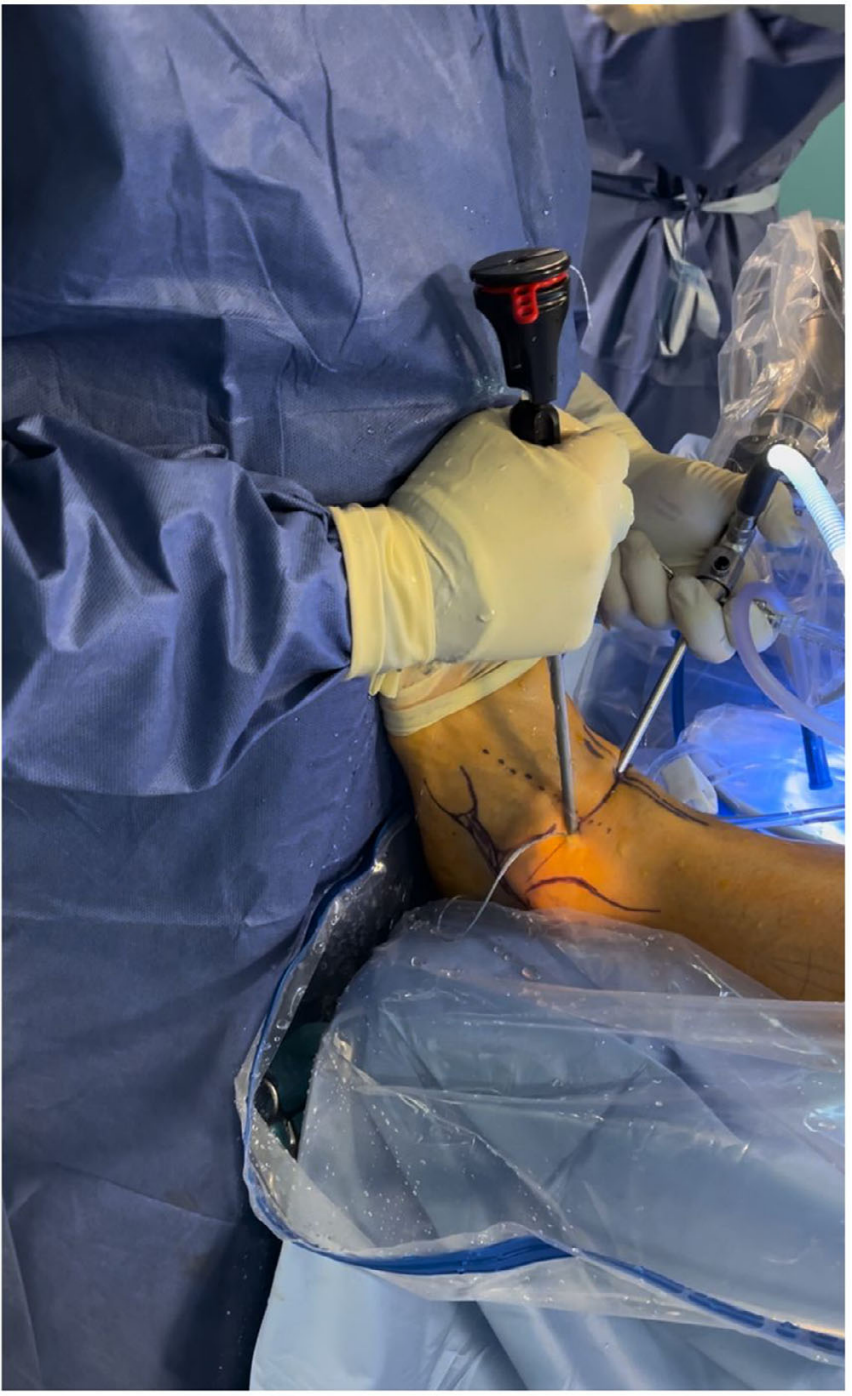
A left ankle is shown with the patient supine. Keeping the guide in place, the second anchor (Arthrex 2.6 mm Knotless FiberTak anchor) is inserted and impacted with tape and hammer (Viewing portal: Anteromedial portal).

The process is repeated for the second anchor. This anchor is placed a little above from the first one but below the anteroinferior tibiofibular ligament insertion. The 2.6‐mm FiberTak guide (Arthrex) is placed and the 2.6‐mm drill makes the bone tunnel. Keeping the guide in place, the anchor (Arthrex 2.6‐mm Knotless FiberTak anchor) is inserted and impacted with gentle taps until the handle is flush with the laser mark in the guide (Figure 7). The red rubber ring is removed, the sutures are unrolled, and the guide and the anchor introducer are removed. As the first anchor, the wires are slowly pulled on to open the intraosseous anchor and secure it.

**FIGURE 8:**
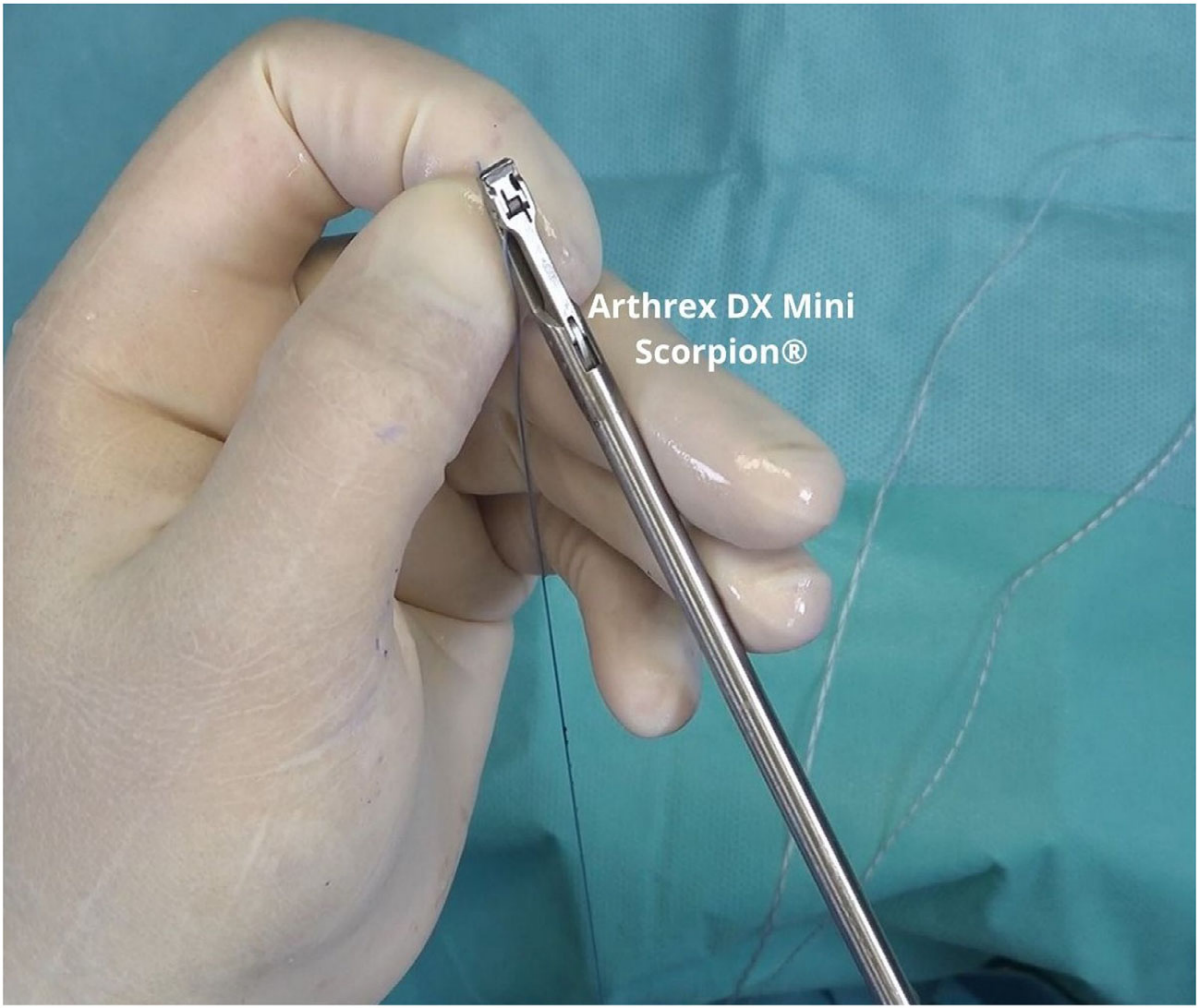
The working blue suture tip is placed on the mini scorpion (Arthrex DX Mini Scorpion).

**FIGURE 9:**
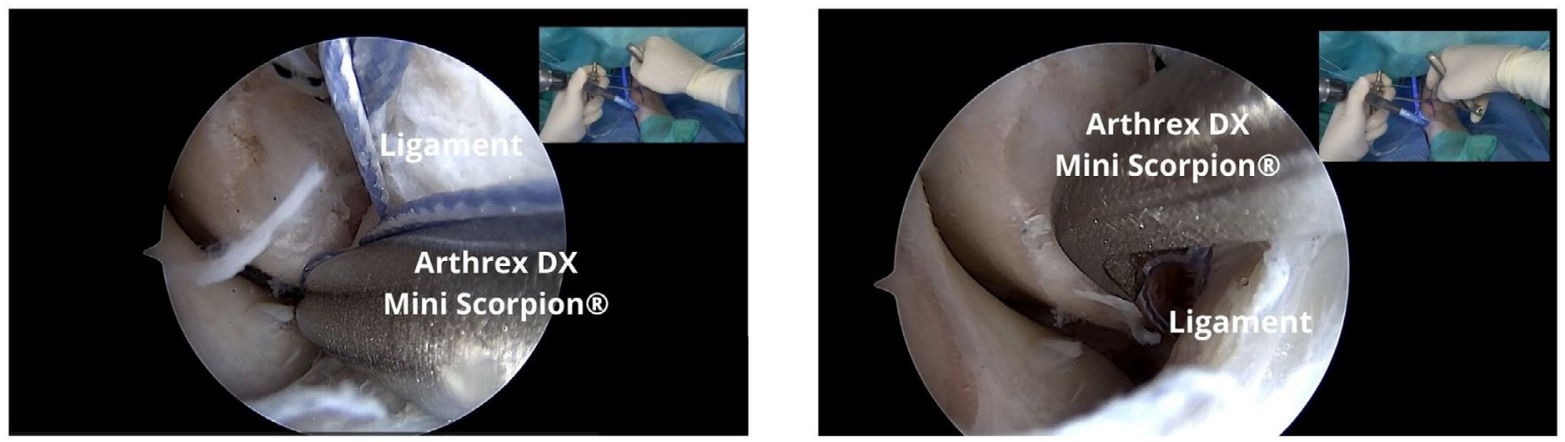
A left ankle is shown with the patient supine. The mini scorpion (Arthrex DX Mini Scorpion) is inverted, and it is passed through the ligament (Viewing portal: Anteromedial portal).

**FIGURE 10:**
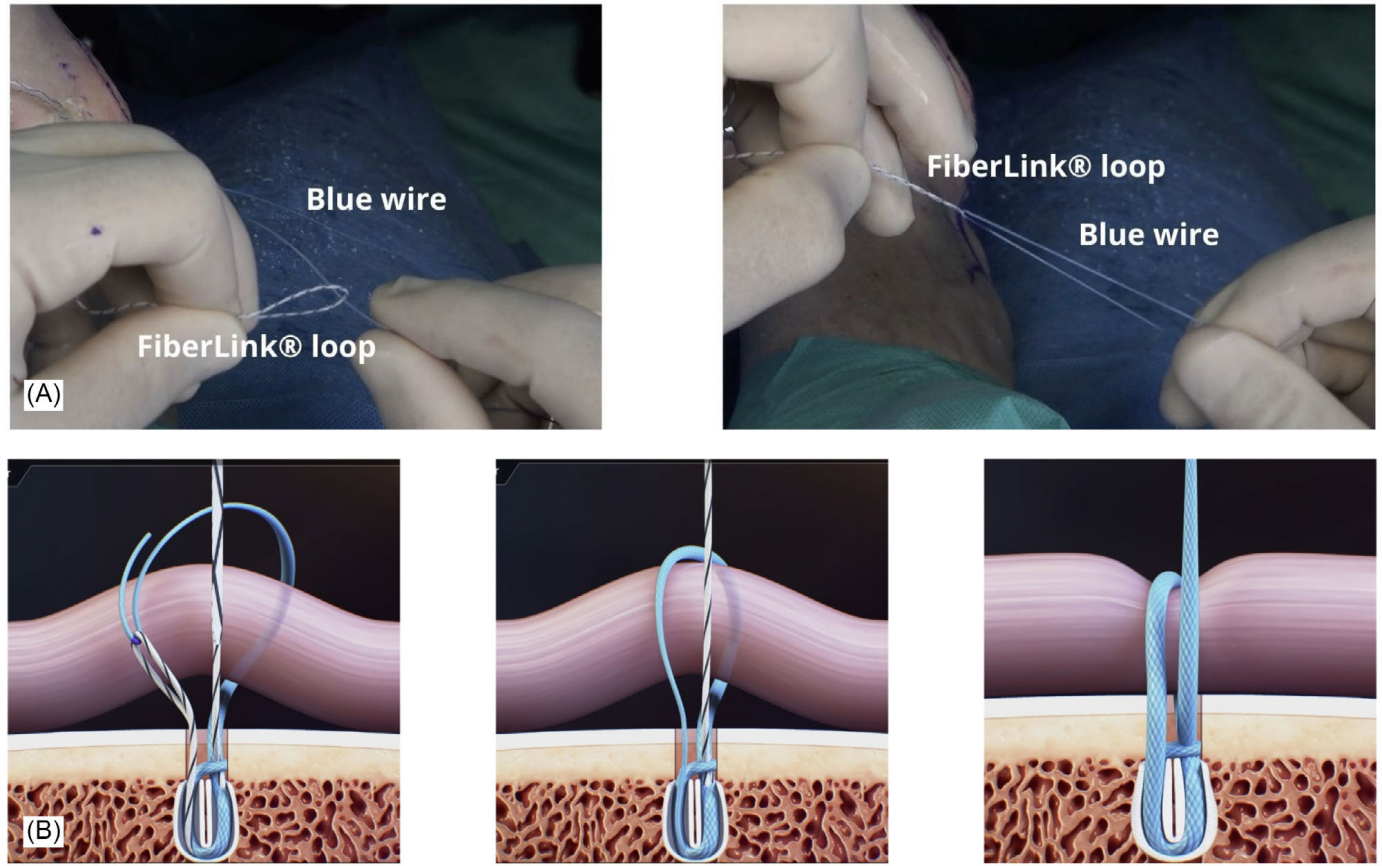
(A) Outside the cannula, pass the blue wire through the looped end of the black and white FiberLink loop to the purple mark on the blue wire. (B) Knotless system (Viewing portal: Anteromedial portal).

**FIGURE 11:**
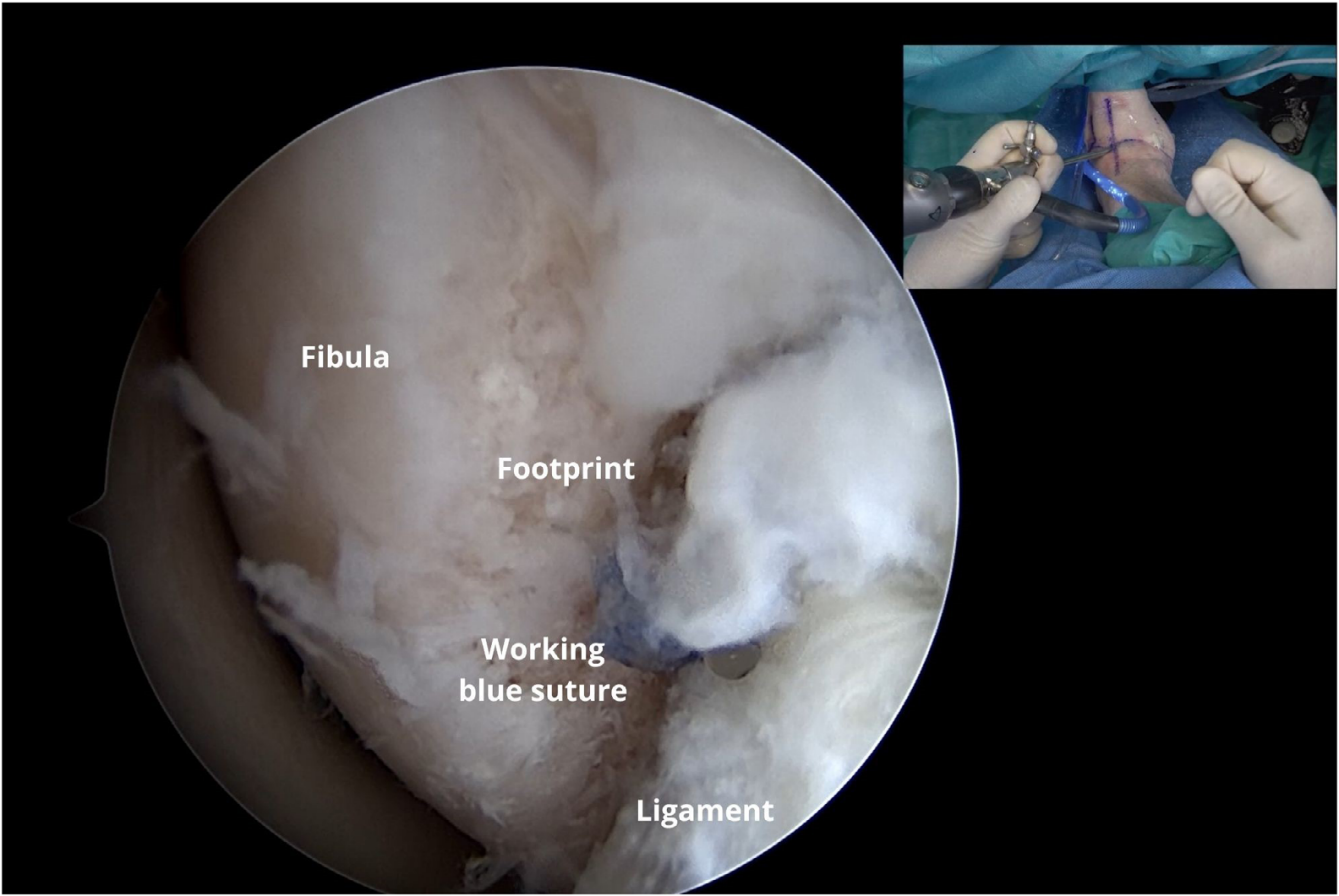
A left ankle is shown with the patient supine. The working blue suture is inside the anchor and the ligament into the footprint, with the foot in a neutral position (Viewing portal: Anteromedial portal).

The blue working suture from the first anchor is loaded onto a DX Mini Scorpion (Arthrex) (Figure 8), and with the MiniScorpion inverted, it is passed through the middle part of the ligament (more closed to the calcaneofibular ligament and to catch the arcade fibers) to pass in an inside‐out manner (Figure 9). The next step is based on the work principles of the anchor knotless system (Figure 10). Outside the cannula, the blue wire is passed through the looped end of the black‐and‐white FiberLink loop (Arthrex), to the purple mark on the blue wire (Figure 10). With a series of light pulls on the FiberLink loop, the working blue suture is pulled inside the anchor, while the ligament is simultaneously being pulled into the footprint, with the foot in a neutral position (Figure 11). The wire is cut with arthroscopic scissors.

**FIGURE 12:**
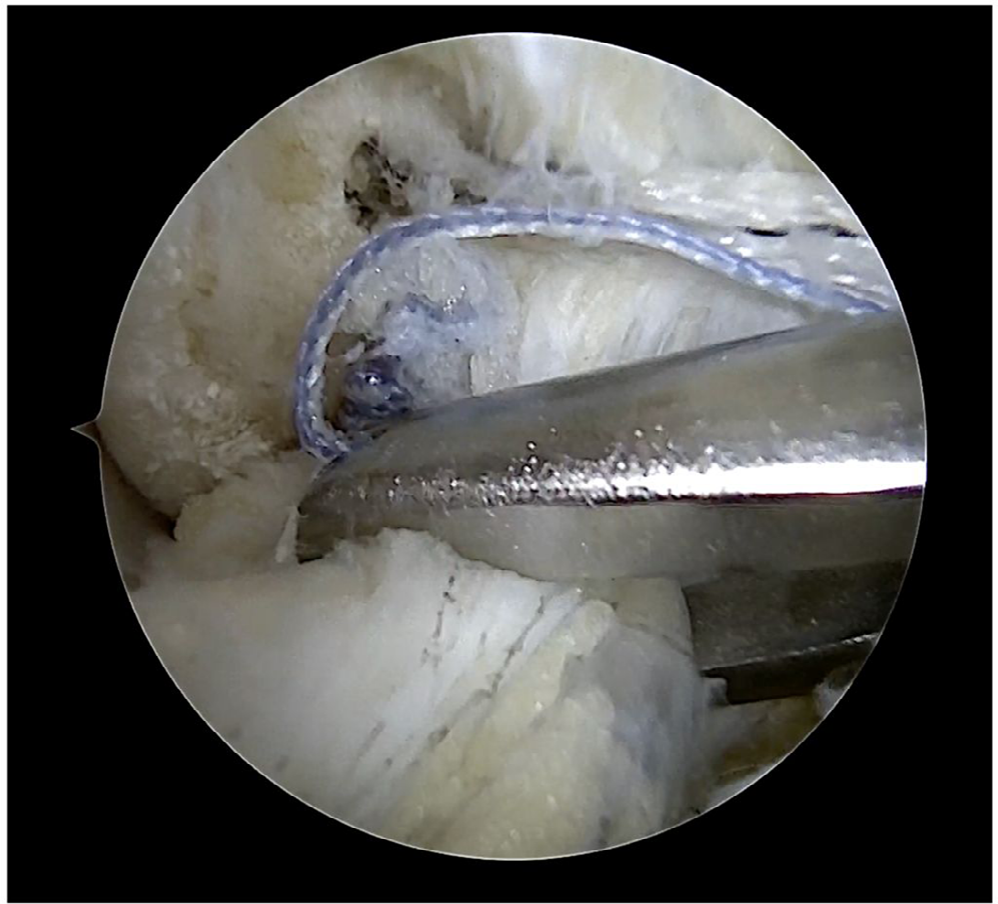
A left ankle is shown with the patient supine. The mini scorpion with the blue suture of the second anchor (Arthrex DX Mini Scorpion) is inverted, and it is passed through the ligament (Viewing portal: Anteromedial portal).

The working blue suture tip of the second anchor is loaded on the Arthrex DX Mini Scorpion and is passed through the most anterior part of the ligament (Figure 12), and the process is repeated as in the first anchor (working blue suture is passed through the looped end of the black‐and‐white FiberLink loop [Arthrex] and pulled inside the anchor). It is tensioned using the same technique, ensuring an anatomic repair as the ligament is being pulled into the footprint.

The ligament's reinsertion tension should be checked with the arthroscopic probe, to ensure that the ligament is properly anchored and not lax.

### Internal Brace Augmentation

The last step is to do an augmentation by the fixation of the internal brace using the 1.7‐mm fibertapes of the second anchor. The two ends of the fibertapes of the second anchor are advanced through the eyelet of the 3.5‐mm SwiveLock anchor.

**FIGURE 13:**
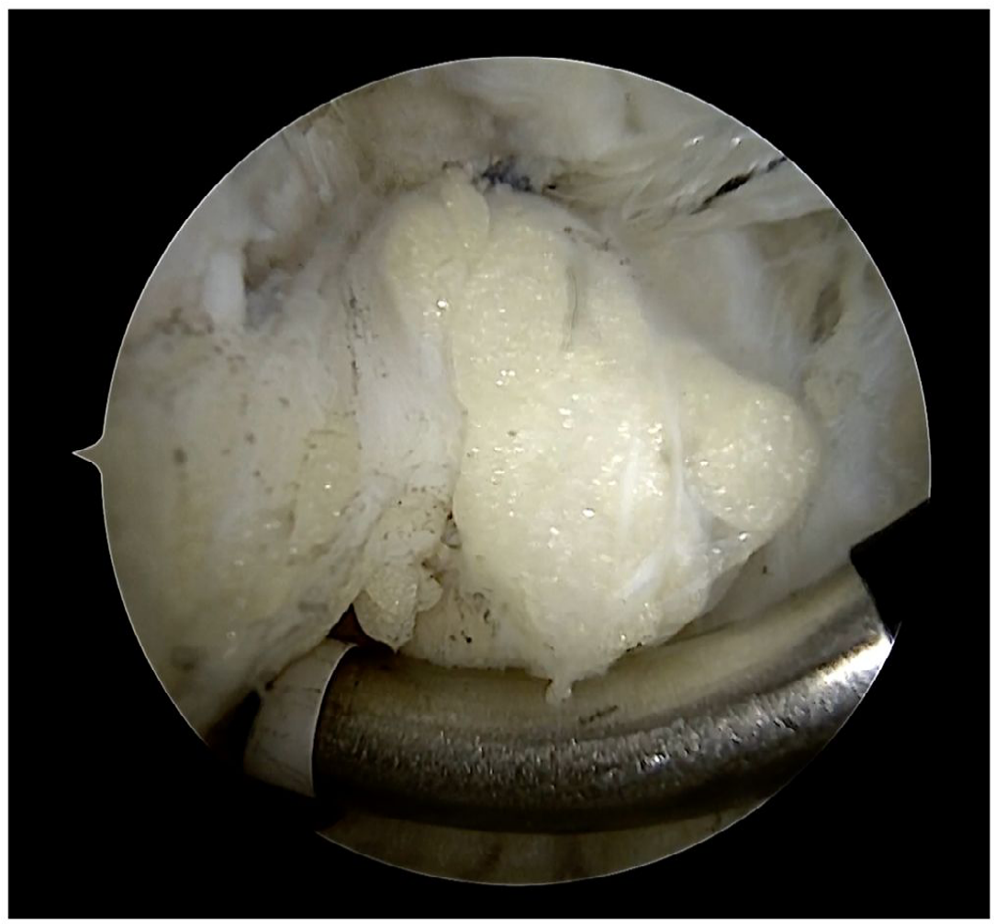
A left ankle is shown with the patient supine. The distal ATFL insertion in the talus is identified. A bone tunnel to the 3.5‐mm SwiveLock is created slightly distal and below to the previous native ATFL insertion (Viewing portal: Anteromedial portal).

Through the working portal, the guide for the 3.5‐mm SwiveLock is inserted. The insertion of the ATFL in the talus is identified. The drill is advanced through the guide and a bone tunnel is created into the talus, slightly distal and below to the previous native ATFL insertion (Figure 13).

**FIGURE 14:**
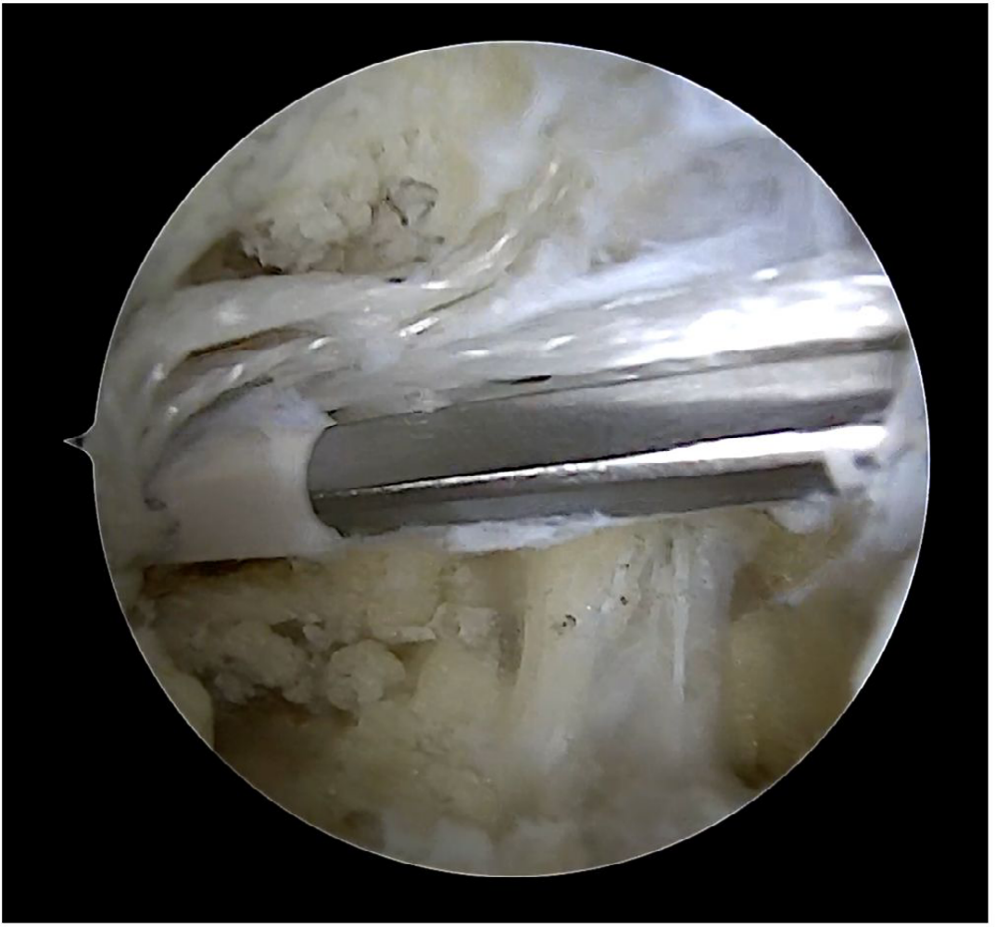
A left ankle is shown with the patient supine. With the foot in neutral inversion/eversion with approximately 10° to 15° of plantar flexion, place the eyelet of the SwiveLock at the entrance of the tunnel and mark the FiberTape suture at the laser line. Slide the eyelet to the line previously marked and insert the anchor into the drilled hole (Viewing portal: Anteromedial portal).

With the foot in neutral inversion/eversion with approximately 10° to 15° of plantar flexion, place the eyelet of the SwiveLock at the entrance of the tunnel and mark the FiberTape suture at the laser line. Slide the eyelet to the line previous marked and insert the anchor into the drilled hole (Figure 14). The anchor is inserted and impacted into the tunnel with gentle taps, and then, hold the square tab in place and turn the pear‐shaped driver, and the anchor is inserted until the end. The tapes are cut with arthroscopic scissors.

With this type of anchor, we do not make sliding knots and, thus, there is less risk of soft‐tissue impingement occurring when tensioning the ligament. Because of this, we can tension the ligament more effectively, and at the same time, there is an internal brace to reinforce and give the biomechanical stability required for high‐demand patients.

### Final Assessment

**FIGURE 15:**
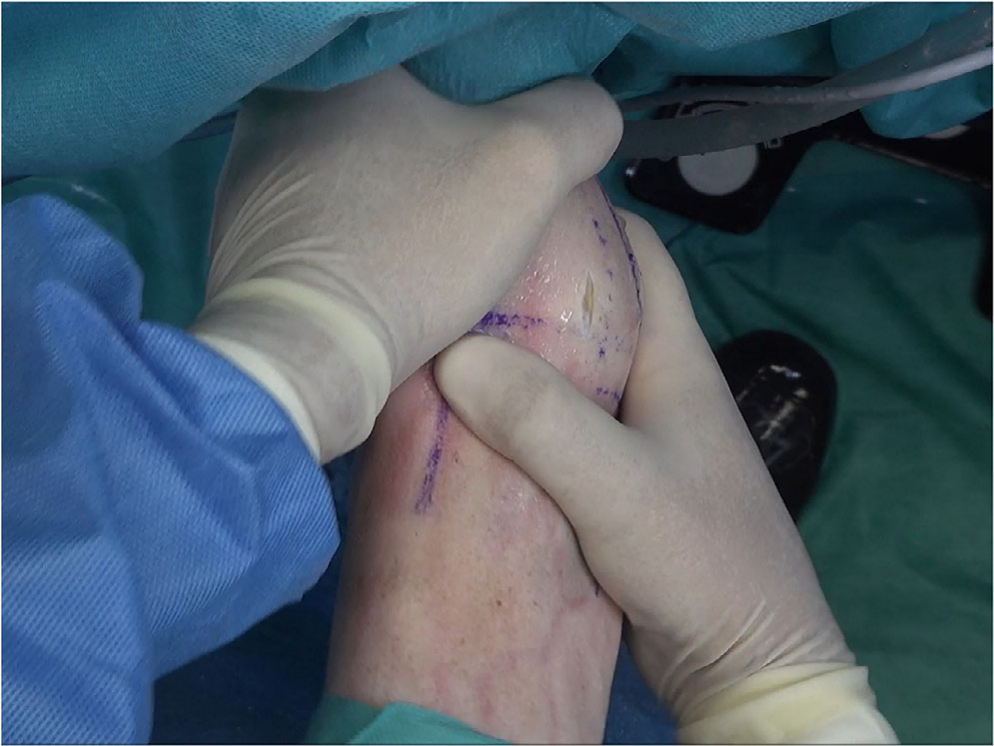
A left ankle is shown with the patient supine. An anterolateral drawer test is performed to confirm joint stability (Viewing portal: Anteromedial portal).

Tensioning is verified by traction testing into the ligament at the arthroscopy and performing the anterior drawer test to confirm stability and ensure appropriate fixation (Figure 15).

### Postoperative Rehabilitation

After the surgery, no immobilization is performed. After 1 week, the patient is put in an ankle brace and can start weight‐bearing according to tolerance, with the aid of crutches. After 2 weeks, the patient is allowed full weight‐bearing, according to tolerance, without the aid of crutches. From 6 weeks onward, the patient can walk without any brace. Resistance exercises should be carried out with a gradual increase in strength (open and closed kinetic chain, as well as functional activities). Return to sport is expected at 12 weeks and full recovery at 4 months.

## DISCUSSION

Although open Broström‐Gould repair is well‐established for CLAI, arthroscopic approaches offer numerous benefits: reduced soft‐tissue disruption, faster recover, and the ability to treat concomitant intra‐articular pathology.^6^, ^7^, ^8^, ^10^, ^11^ The described technique herein combines these advantages with modern innovations in suture anchor technology.

Knotless suture anchors eliminate issues related to knot bulk and soft‐tissue irritation, while providing stronger and more reproducible fixation compared with traditional constructs.^12^, ^13^, ^14^ Moreover, the addition of an internal brace reinforces the repair, potentially reducing reinjury risk in athletes while also enabling earlier rehabilitation.

When the residual tissue of a torn ATFL is of insufficient quality to support primary repair, ligament reconstruction is generally indicated. However, biomechanical studies support anatomic repair over reconstruction whenever feasible, as it better preserves native joint mechanics and proprioceptive function.^15^, ^16^ Only through repair can the native histological properties of the ligament—including mechanoreceptors critical for proprioception—be maintained.

All‐inside arthroscopic ATFL repair with nonabsorbable suture augmentation offers the dual advantage of preserving the native ligament tissue while reinforcing the construct. This technique enables early mobilization and eliminates the need for prolonged immobilization.^17^

Complications such as superficial peroneal nerve injury remain a concern and highlight the importance of careful preoperative planning and surgeon experience (Table 2).

**TABLE 2:** Advantages, Risks, and Limitations of the Presented Technique

Advantages	Risk and Limitations
Allow the restoration of the ATFL's stability	Be careful not to damage the superficial peroneal nerve
To create a bone tunnel, minimal bone is removed	If not tensioned in the correct position can lead to stiffness of the ankle
The fact that no sliding knots are made reduces the risk of having soft‐tissue impingement when tensioning the ligament and, thus, it can tension the ligament more effectively	
Can be used simultaneously with other bony procedures	
Enables early mobilization and eliminates the need for prolonged immobilization	

ATFL, anterior talofibular ligament.

Nevertheless, this technique offers a comprehensive, anatomic, and minimally invasive solution for patients with CLAI.

## DISCLOSURES

The authors (B.S.P., F.S.M., P.D.) declare that they have no known competing financial interests or personal relationships that could have appeared to influence the work reported in this article.
